# Targeting phosphatidylserine synergizes with immune checkpoint blockade by inducing de novo tumor specific immunity

**DOI:** 10.1186/2051-1426-3-S2-P358

**Published:** 2015-11-04

**Authors:** Xianming Huang, Jian Gong, Dan Ye, Van Nguyen, Michael Gray, Steven King, Jeff Hutchins, Rolf Brekken, Bruce Freimark

**Affiliations:** 1 University of Texas, Southwestern Medical center at Dallas, Dallas, TX, USA; 2 Peregrine Pharmaceuticals Inc., Tustin, CA, USA

## Background

Extensive studies have demonstrated that the inside-out membrane phospholipid phosphatidylserine (PS) actively drives global immunosuppression in the tumor microenvironment and is a major contributor to tumor resistance to immune checkpoint blockade. We have shown that PS targeting antibodies can re-program the tumor microenvironment from immunosuppressive to immune potentiating by reducing the number of myeloid-derived suppressor cells (MDSCs), repolarizing tumor associated macrophages from M2 to M1 and by promoting the maturation of dendritic cells. We have found that PS targeting antibodies synergize with and significantly enhance the therapeutic efficacy of immune checkpoint blockade in multiple tumor models. In the present study, we examined the effect of combination of a PS targeting antibody and anti-PD-1 or anti-CTLA-4 on tumor-specific CD8 T cell immunity.

## Methods

B16 tumor-bearing mice were treated weekly i.p. at 5 mg/kg with each antibody or combination. Splenocytes were harvested after three treatments. The number of tumor-specific IFNg-producing splenocytes was evaluated by ELISPOT in the presence or absence of irradiated B16 tumor cells. Tumors were harvested and single cell suspensions were obtained and immune profiled by FACS.

## Results

In the absence of B16 tumor cell stimulation, the combination of ch1N11 and anti-PD-1 resulted in the highest number of IFNg Elispots (109±25), significantly better than anti-CTLA-4 (29.4±4, p < 0.005), ch1N11 (16.6±3.2, p < 0.001), anti-PD-1 (41.6±5.5, p < 0.001), and ch1N11+anti-CTLA4 (73.6±13.9, p < 0.05); in addition, the combination of ch1N11 and anti-CTLA-4 was significantly better than anti-CTLA-4 alone (29.4±4.2, p < 0.01), indicating that there were significantly more functional T cells in the spleens of mice treated with combination therapy. Importantly, stimulation with irradiated B16 tumor cells resulted in robust induction of IFNg production from splenocytes harvested from mice treated with ch1N11 and anti-PD-1. Tumor cell stimulation resulted in a >2-fold increase in IFNg Elispots (198± 39 vs 70±5, p < 0.01), which was also thirteen-fold higher than that of control group (15±2.1, p < 0.001). These data demonstrate that combination treatment induced significantly more tumor specific T cells. FACS analysis of tumor infiltrating lymphocytes (TILs) indicated that combination of antibody-mediated blockade of PS and PD1 significantly enhanced effector function of tumor infiltrating CD8+ T cells, as demonstrated by significant increases in TILs producing IFN-g, TNFα, IL-2, granzyme B, and Ki-67.

## Conclusions

PS targeting antibodies synergize with immune checkpoint blockade to induce strong tumor specific CD8 T cell immunity.

